# Temporal dynamics in animal community assembly during post-logging succession in boreal forest

**DOI:** 10.1371/journal.pone.0204445

**Published:** 2018-09-20

**Authors:** Hélène Le Borgne, Christian Hébert, Angélique Dupuch, Orphé Bichet, David Pinaud, Daniel Fortin

**Affiliations:** 1 Chaire de recherche industrielle CRSNG-Université Laval en sylviculture et faune, Département de biologie, Pavillon Alexandre-Vachon, Université Laval, Québec, QC, Canada; 2 Natural Resources Canada, Canadian Forest Service, Laurentian Forestry Centre, Stn. Sainte-Foy, Québec, Canada; University of Split, Faculty of science, CROATIA

## Abstract

Species assemblages can result from deterministic processes, such as niche differentiation and interspecific interactions, and from stochastic processes, such as random colonisation and extinction events. Although changes in animal communities following disturbances have been widely examined, few studies have investigated the mechanisms structuring communities during ecological succession. We assessed the impact of logging on small mammal and beetle assemblages in landscapes dominated by old-growth boreal forests. Our objectives were to 1) characterize variations in communities during the first 66 years of post-harvest forest succession, 2) determine if there are non-random patterns of species co-occurrence (i.e., deterministic processes), and if there are, 3) establish whether non-random co-occurrences are best explained by habitat attributes or by interspecific interactions. We captured small mammals and beetles along a gradient of forest succession (5–66 years) and in old-growth forest, and characterized key vegetation attributes. First, we tested whether community compositions in clear-cut stands became similar to those in natural stands after 66 years. We then used null models, which were either unconstrained or constrained by habitat attributes, to address the last two objectives and distinguish effects of vegetation attributes from interspecific interactions on community assembly. We showed that beetle assemblages differed in stands 21–30 years post-harvest compared to old-growth forests. In contrast, harvesting did not influence the composition of small mammal communities. Overall, our results suggest that community assembly during forest succession is driven by both stochastic and deterministic processes, the latter being linked to interspecific interactions more strongly than to vegetation attributes.

## Introduction

Natural and anthropogenic disturbances can alter animal species assemblages by modifying biotic and abiotic habitat features for decades [1–4]. While temporal changes in animal populations and communities following disturbances such as fire [5,6] or timber harvesting [7,8] have been widely described, few studies have identified how the processes structuring communities are affected [9]. With the increasing rate at which natural habitats are being modified, there is a need to better understand how disturbances alter ecosystem properties and processes, including community assembly.

Species assemblages can develop from deterministic processes, such as niche differentiation, predation, competition, and differential responses of species to their environment [1,10,11], from stochastic processes that are linked to colonization, extinction and speciation [12], or from a combination of both [13,14]. The relative effects of deterministic and stochastic processes in structuring assemblages may vary with habitat alteration [15], followed by ecological succession [16]. For example, the competitive effects of the dominant ant species on the abundance of other species increase with time since disturbance in Fennoscandian boreal forest [17].

Deterministic and stochastic processes structuring community assembly can be revealed by null-model analysis, which compares species co-occurrence that is observed with random expectations of co-occurrence patterns [18–20]. If stochastic processes drive species assemblages, species co-occurrences should not differ from a random distribution. Conversely, if deterministic processes structure communities, individual species should co-occur more or less often than what would be expected randomly [9]. In typical null models, evidence for non-random patterns does not directly identify the factors that are responsible, such as competition or environmental suitability. Null models including habitat constraints can thus be used to identify the processes underlying patterns of species distribution and to distinguish between the effects of habitat and interspecific interactions [19].

Forests are among terrestrial habitats harbouring the greatest species richness [21]. Forest ecosystems are being transformed at an increasing pace by human activities with consequences on species assemblages that remain largely elusive. For example, while logging is among human activities having the strongest impact on animal communities [22], most studies on animal community assembly have focused solely on short-term effects of timber harvesting [23,24]. As a result, time and processes that are responsible for community recovery during post-logging forest succession remain unclear [25,26]. Such information is relevant to a broad range of scientific fields, from community ecology to biodiversity conservation and forest management. For example, given the conservation objective of maintaining biodiversity, the time required for animal species to return to their pre-disturbance assemblages could be among the criteria used to determine harvesting rotation length. If rotations were too short, then animal assemblages would have insufficient time to return to their typical states; this can result in subtle changes in animal communities, first characterized by the loss of barely mobile species that are typically found in old-growth stages.

Studies linking habitat characteristics and animal species diversity typically have focused on birds, which are influenced by landscape scale variables [27]. Yet, small mammals and insects also play important roles in forest ecosystems, and they can be influenced by stand scale attributes. Indeed, small mammals are prey for several mammalian and avian predators, and they disseminate seeds and spores, decompose organic matter and litter, and consume a broad-range of plant species [28,29]. Insects are the most diverse group of organisms, and they affect various ecosystem processes, such as pollination and decomposition, through their high functional diversity (as saproxylics, herbivores, predators, and fungivores, see, e.g., [30]). Moreover, numerous studies have emphasised the sensitivity of both small mammals and insects to forest management, with disturbed forests often supporting assemblages that differ from those found in old-growth forests [28,29,31–33].

The purpose of this study was to identify the key processes shaping small mammal and beetle communities during ecological succession after clear-cutting in old-growth boreal forest. We focused upon these questions: 1) Had animal community composition of logged forests recovered after 66 years (i.e., age of the oldest harvested stands sampled in our study) and is it comparable to that found in old-growth forests? 2) Do communities assemble through deterministic processes (i.e., to form non-random associations) during forest succession following clear-cutting, and if they do, 3) are they influenced more strongly by habitat attributes or interspecific interactions? To answer these questions, we characterized species composition patterns along a boreal forest succession, and assessed non-random patterns based on null model analyses incorporating different habitat constraints.

## Materials and methods

### Study area

**Fig 1:**
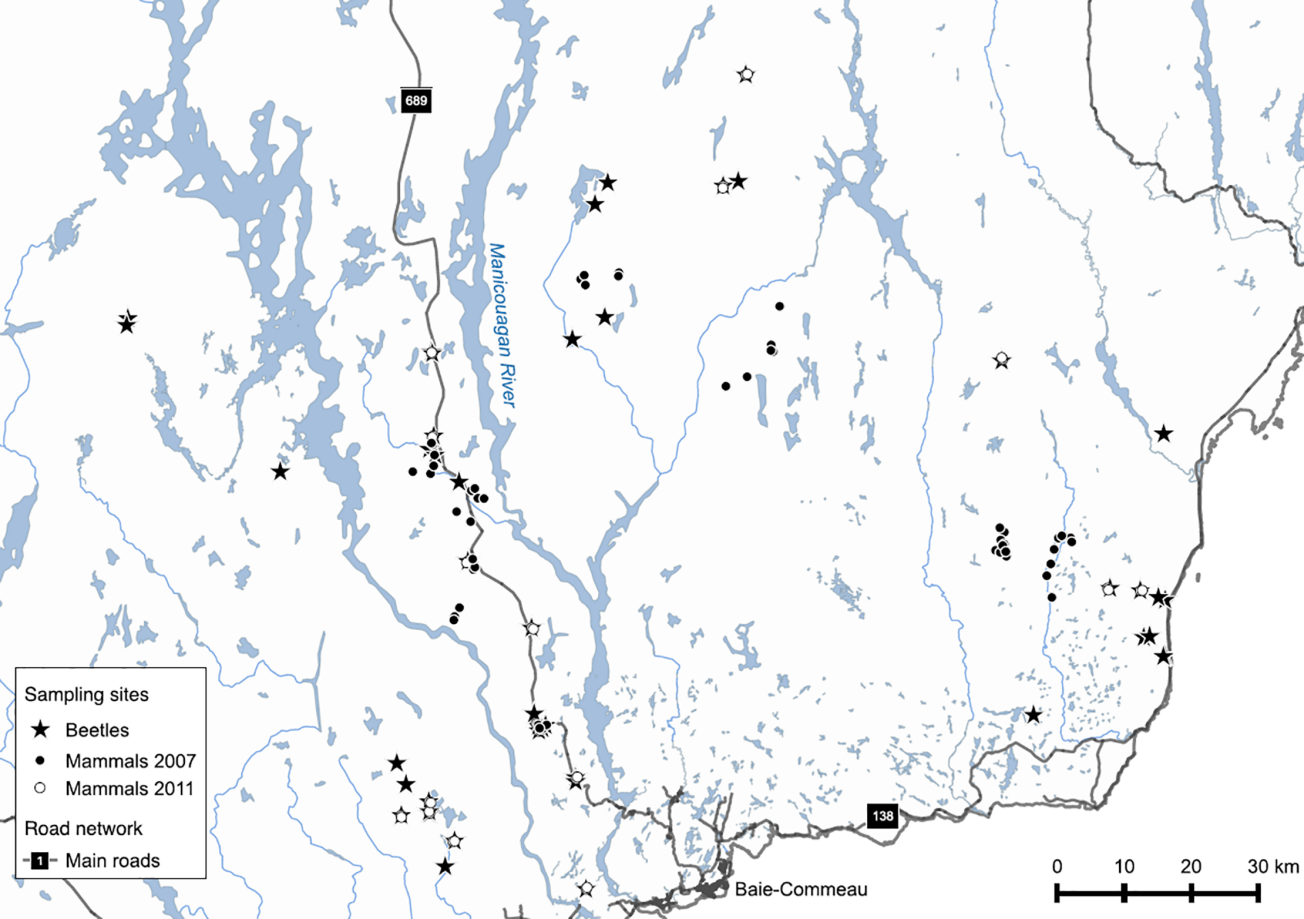
Study area and the locations of sampling sites. Locations of sampling sites for small mammals (from both years 2007 and 2011) and beetles within the study area, in the boreal forest of the Côte-Nord region, Québec, Canada.

The study was conducted in the boreal forest of northeastern Québec, Canada (Fig 1). The climate of this region is humid and cold, with a mean annual precipitation of 1014 mm and an annual mean temperature of 1.5°C [34]. The fire cycle is long, exceeding 270 years, which explains the large proportion of irregular old-growth stands in the region [35]. With long fire cycle, fine-scale disturbances such as windthrows or insects and diseases that kill old trees largely shape forest attributes, with stands often developing this irregular structure [36]. The old growth forest within the region is composed of old-growth stands that are dominated by black spruce (*Picea mariana*) and balsam fir (*Abies balsamea*), with scattered paper birch (*Betula papyrifera*) and trembling aspen (*Populus tremuloides*) [34].

To evaluate how species assemblages vary during post-harvest forest succession, we selected cut stands (5- to 66-years-old) from archives of forest product companies. Clear-cutting was mainly used in the study area until 1996, at which point another logging practice (i.e. CPRS for “Coupe avec Protection de la Régénération et des Sols”) was implement by only harvesting trees with a diameter at breast height >9 cm while protecting soils and regeneration [37,38]. To minimise the risk that temporal changes in silvicultural practices influenced our conclusions, we only selected post-CPRS stands if they had low tree retention (i.e. < 10% of the basal area) so that they were comparable in structure to clear-cutting. We also selected uncut stands that were representative of spruce–moss boreal forest (dominated by black spruce and balsam fir), i.e. old-growth forest > 120-years-old because they are similar in terms of habitat characteristics with irregular vertical and horizontal structures [39]. We surveyed only stands that met the following criteria: i) had a minimum area of 6 ha (250 × 250 m); ii) streams and roads were absent from the sites; iii) cover was dominated by conifers (black spruce and balsam fir); and iv) only natural regeneration took place (no thinning or planting). Forest and logging information came from the third decadal forest inventory program (Eco-forestry Information System, Ministère des Ressources naturelles et de la Faune du Québec), together with information from the forestry companies that were working in the area (i.e., Resolute Forest Products and Arbec Forest Products Inc.), which were stored in a geographic information system that was managed with ArcGIS 9.2 (ESRI, Redlands, CA, USA).

### Small mammal sampling

We sampled 53 sites in 2007 (37 logged stands ranging from 5- to 62-years-old, and 16 old-growth forests, Fig 1) and 34 sites in 2011 (17 logged stands ranging from 5- to 66-years-old and 17 old-growth forests) between June and September of each year. The logged sites were spaced by at least 1 km to survey independent populations (i.e., logged sites were distant by much more than the typical dispersal distances of small mammals [40], and to avoid spatial autocorrelation issues (range of variograms < 1 km, see[2]. In 2007, small mammals were sampled at each site using a grid of 7 × 7 live traps (7.7 × 8.8 × 23.0 cm; Sherman Traps, Tallahassee, FL), with a distance of 10 m between traps. The beginning of the grid was placed > 120 m from stand edge. In 2011, we sampled small mammals using live traps placed every 10 m along each of two parallel transects (100 m apart), running perpendicular from and centred on the stand edge shared by the adjacent cut and uncut stands. Transects extended 150 m into each stand (for a total of 300 m); thus, 30 traps were set up in each stand. For both sampling years, a piece of apple was placed in each trap with peanut butter and some cotton wool. Traps were left out for three consecutive days, and were inspected and reset every day at dawn. Each captured individual was identified and ear-tagged with a unique number (Style 1005–1, National Band & Tag, Newport, KY). The experimental design differed between the two years because the two studies had different specific objectives, but they were located in the same study area (Fig 1). Combining the two datasets increased the number of replicates for each stand age, while also encompassing a broader spatial domain. Ultimately, this approach provided a stronger appraisal of community composition at each successional stage along the chronosequence. All animal handling and experimentation were approved by the University Laval Animal Welfare Committee, and the Ministère des Ressources naturelles et de la Faune du Québec has issued research permit.

### Beetle sampling

In each stand, beetles were sampled from 30 May to 21 August 2011 when most insects were active in this boreal region [41], in 35 cut stands (4- to 66-years-old) and 10 old-growth forests (34 sites were in common with small mammal sampling) located at least 1 km from each other (Fig 1) to avoid potential spatial autocorrelation issues (see also [2]). We used two different types of traps: flight-interception traps for flying beetles, and pitfall traps for ground-dwelling beetles. We positioned the multi-directional flight-interception trap 0.5–1 m above the ground at the centre of each site. The trap was constructed using four 15 × 40 cm panels (two made of Plexiglas and two of mosquito netting) that were mounted in a cross pattern, along a 10-cm diameter black cylinder, with two funnels located above and below the cylinder that led to collecting vials [33]. Four pitfall traps were placed in a cross design seven metres from the centre and 10 m from one another [2]. Traps were partly filled with 40% ethanol solution with traces of household vinegar (5% acetic acid) to kill and preserve all insects. Traps were visited every 3 weeks and samples were preserved in 70% ethanol. Most specimens were identified at the species (92.6%) or genus (6.9%) level, depending on the available taxonomic tools. Few specimens (< 1%) were identified at the tribe or family level.

### Vegetation sampling

In each stand, vegetation was characterized both in 2007 and 2011 at sampling points located > 120 m from the forest edge. In 2007, vegetation was characterized at three different sampling points (42 m from one another) within the capture grid of small mammals, whereas in 2011 we used the location of the flight-interception trap as sampling point. In 2007, ground cover (in percent) of dead wood and fallen branches, rocks, mosses, lichens, graminoids, herbs and shrubs were visually estimated in three 1-m^2^ plots that were randomly located around each sampling point. In 2011, percent cover was estimated in four 4-m^2^ plots located 2 m to the North, South, East and West of the sampling point. The DBH of every living and dead tree (≥ 9 cm) was recorded in either 200 or 400 m^2^ circular plots (depending upon tree density) that were centered on each sampling point, and tallied in 2-cm DBH classes according to species and status (dead/alive) for both years. The quantity of coarse woody debris (CWD; length ≥ 1 m and diameter at both ends ≥ 9 cm) that was lying on the ground was also recorded in the 200-m^2^ (or 400 m^2^) plots for 2007, and along two perpendicular 11.28 m transects (i.e., the radius of a 400-m^2^ circle) that was centered on the circular plot for 2011.

We calculated several indices to test the influence of habitat heterogeneity on species assemblages. Structural heterogeneity in each stand was described with five indices: (1) the number of diameter classes (2-cm classes) for standing trees, combining live and dead individuals; (2) the amount of CWD; and (3) time-since-logging (stand age). We also estimated compositional heterogeneity through (4) the number of tree and shrub species, and (5) the dominant tree species in the stand. We chose these variables because they can influence small mammal and beetle species distributions or community structures [1,2,29,42]. Indices 1, 2 and 4 were each divided into 3 classes with about the same number of sites in each category, respectively representing low, medium and high values. Because the methodology for data collection differed slightly between 2007 and 2011, CWD estimates were not readily comparable between 2007 and 2011. In order to use a single variable summarizing CWD variation among sites, we transformed the quantity of CWD into a binary variable, where the value was either higher (1) or lower (0) than the median value in each year (which was different in 2007 and 2011). We divided the stand age into five age-classes: 4–20, 21–30, 31–50, 51–70 and > 120 years since logging, which provided enough sites in each category to allow interclass comparisons (small mammals with 9 to 33 sites per class; beetles with 7 to 10 sites per class).

Age classes corresponded to establishment stage (4- to 20-years-old), aggradation stage (early: 21- to 30-; mid: 31- to 50-; late: 51- to 70-years-old) and old-growth forest structural stages (> 120 years-old), respectively, as described by successional stages in the North American boreal forest that were determined by [43]. The last index representing the dominant tree species in the stand is composed of three mutually exclusive classes: stands that were dominated by either spruce or fir species (species representing more than 60% of basal area); and stands co-dominated by both spruce and fir species (species representing 40 to 60% of basal area).

### Statistical analyses

#### Species co-occurrence database

Species that occurred in <10% or >90% of the sites were excluded from the analysis (e.g., red-backed voles, *Myodes gapperi*), because rare or ubiquitous species provided limited information on habitat preferences and factors influencing species co-occurrences [1,41]. We considered five small mammal species or groups: red squirrel (*Tamiasciurus hudsonicus*), eastern chipmunk (*Tamias striatus*), shrews (*Sorex* spp.), deer mouse (*Peromiscus maniculatus*), and a “lemming and rock vole” category that included both rock vole (*Microtus chrotorrhinus*) and southern bog lemming (*Synaptomys cooperi*). We combined these two species, even if they were caught in less than 10% of the sites because they have similar food preferences (e.g., herbivorous and fungivorous) and habitat (e.g., young forest and humid areas; [44] and they exceeded the 10% threshold when combined into a single class.

#### Analyses of species composition patterns

We examined how species assemblages changed among four age classes characterising post-harvest succession (4 to 20, 21 to 30, 31 to 50, and 51 to 70-years-old) and compared these assemblages with those found in old-growth forest, which is the successional stage of reference in our study. For small mammals, data from 2007 and 2011 were combined to provide a more general assessment of changes in species assemblages during post-harvest succession. We examined the compositional differences (β diversity) among forest age classes using a Permutational Multivariate Analysis of Variance (PERMANOVA) and Permutational Analysis of Multivariate Dispersions (PERMDISP) based on a semi-metric distance matrix calculated from Sørensen Dissimilarity indices in R (R Development Core Team 2011, version 2.15.0). We used respectively the *adonis* and *betadisper* functions, from the *vegan* package, to test differences in species composition estimated with the Sørensen Dissimilarity index for small mammals, ground beetles and flying beetles among forest age classes. PERMANOVA (based on 999 permutations of the data) tested for differences in assemblages between treatments (i.e., forest age classes). For small mammals, data permutations were done individually for each year (2007 or 2011) to avoid mixing data collected with different sampling protocols. When PERMANOVA detected significant differences among age classes, we carried out pairwise contrasts between each post-harvest age class and our successional stage of reference, old-growth forest. *P*-values were adjusted for multiple comparisons using Holm’s sequential Bonferroni procedure. A significant result in PERMANOVA may indicate assemblage differences across treatments, differences in within-treatment dispersion (i.e., heterogeneity of multivariate dispersion within groups), or both [45]. To properly interpret the PERMANOVA results, we used PERMDISP when PERMANOVA was significant, which tests for homogeneity in multivariate dispersion between age classes. When PERMDISP detected significant differences between age classes, we used Tukey HSD to contrast all age classes with old-growth forest. To visualise the compositional differences between age classes and compositional dispersion of treatments from the community centroids, we used principal coordinates ordination plots (also known as Classical multidimensional scaling, MDS) based upon the species composition dissimilarity matrix. We used an 80% confidence ellipse, as it enclosed most sites within their respective groups, and provided adequate graphical representation of their positions relative to other groups. Stands in which no small mammals were captured were removed from the analyses (PERMANOVA: *N* = 12). We verified the absence of autocorrelation in our small mammal data from 2011 due to the paired experimental design with a Mantel test (*mantel* function from the *vegan* package) between the species composition dissimilarity matrix and the Euclidian distance matrix among sites (r = 0.047, p = 0.237).

We also determined how the abundance of the ubiquitous red-backed vole varied during post-harvest succession using linear mixed models that were performed with the package *nlme* (R Development Core Team 2011). We took into account differences in experimental design between years by including site nested within year as random intercepts. Red-backed vole abundance and time-since-logging were log-transformed to normalise the variables prior to analysis.

#### Null model analysis of species co-occurrence patterns

We used null model analysis to test whether observed species pairwise co-occurrence patterns were more strongly influenced by deterministic or stochastic processes (if not significantly different from those obtained by randomising species distributions). We followed the procedure of Peres-Neto et al. [19] and Azeria et al. [1], and used two sets of null models. The first one considers only the occurrence of species in the distribution matrix (i.e., unconstrained null models) to quantify whether observed pairwise co-occurrence patterns differed from random expectations. The second null model also considers the potential influence of species’ habitat preferences (i.e., habitat-constrained null models). The sign and significance of associations between pairs of species detected under unconstrained versus habitat-constrained null models were then used to evaluate the potential role of habitat and interspecific interactions on species co-occurrence.

For unconstrained null models, we used two types of models, i.e., fixed-fixed (FF) and fixed-equiprobable (FE). Both FF and FE models maintain the same overall species occurrence frequencies as those contained in the observed data. While FF also maintains species incidence and the total number of species at each site, FE considers sites to be colonised equiprobably (i.e., sites are equally suitable). FF null models tend to detect relatively more negative associations, whereas FE null models tend to detect more positive co-occurrences [1]. Therefore, we used FF and FE null models to identify segregated and aggregated pairs of species, respectively. Both types of null models provided relatively low risk for type I and II errors [18]. If the number of segregated and aggregated pairs of species was higher in the observed distribution matrix than in the unconstrained null models, it would indicate that species communities were most strongly driven by non-random processes.

The habitat-constrained null models were also based upon FE and FF null models, but incorporated habitat constraints, i.e., habitat affinities of species estimated from our dataset (in accordance with [1,19]), which in our analyses were the discrete categories of our five heterogeneity indices. These models were constructed by assigning each site to a habitat class, and randomisations were performed separately within each subset of sites. As a result, the habitat-constrained null models maintained the species frequency within each habitat type and controlled either for species incidence (HCFE models), or for both species incidence and site species richness (HCFF models).

To differentiate between the role of habitat and interspecific interactions in species co-occurrences, we followed the approach of Peres-Neto et al. [19], which compares the sign and significance of the associations that are detected under unconstrained versus habitat-constrained null models (i.e., FF vs HCFF, and FE vs HCFE). For example, if unconstrained models detected a significant species pairwise association but constrained models detected a non-significant one, the co-occurrence could be attributed to similarities (if positive) or differences (if negative) in habitat preferences. Alternatively, if an association was significant in both unconstrained and constrained models, the response could likely be attributed to interspecific interactions. Similarly, an association can also be attributed to interspecific interactions if it was significant in constrained models but non-significant in unconstrained models. Further details regarding the interpretation of the outcomes are outlined in Appendix 1 of [1], and in [19].

We generated 1000 random matrices for each null model with the *vegan* package in R and used the Sørensen Dissimilarity index to evaluate the strength of species pair co-occurrences (for more details see S1 Appendix). We determined whether each pairwise association was an aggregation or segregation by counting the number of null matrices in which association value was lower than, higher than, or identical to those that were obtained from the observed co-occurrence data. Associations were considered statistically significant if the observed Sørensen index lay outside of the 95% distribution of the index values that had been obtained from the 1000 simulated null matrices.

## Results

### Variation in small mammal and beetle communities during forest succession

**Fig 2:**
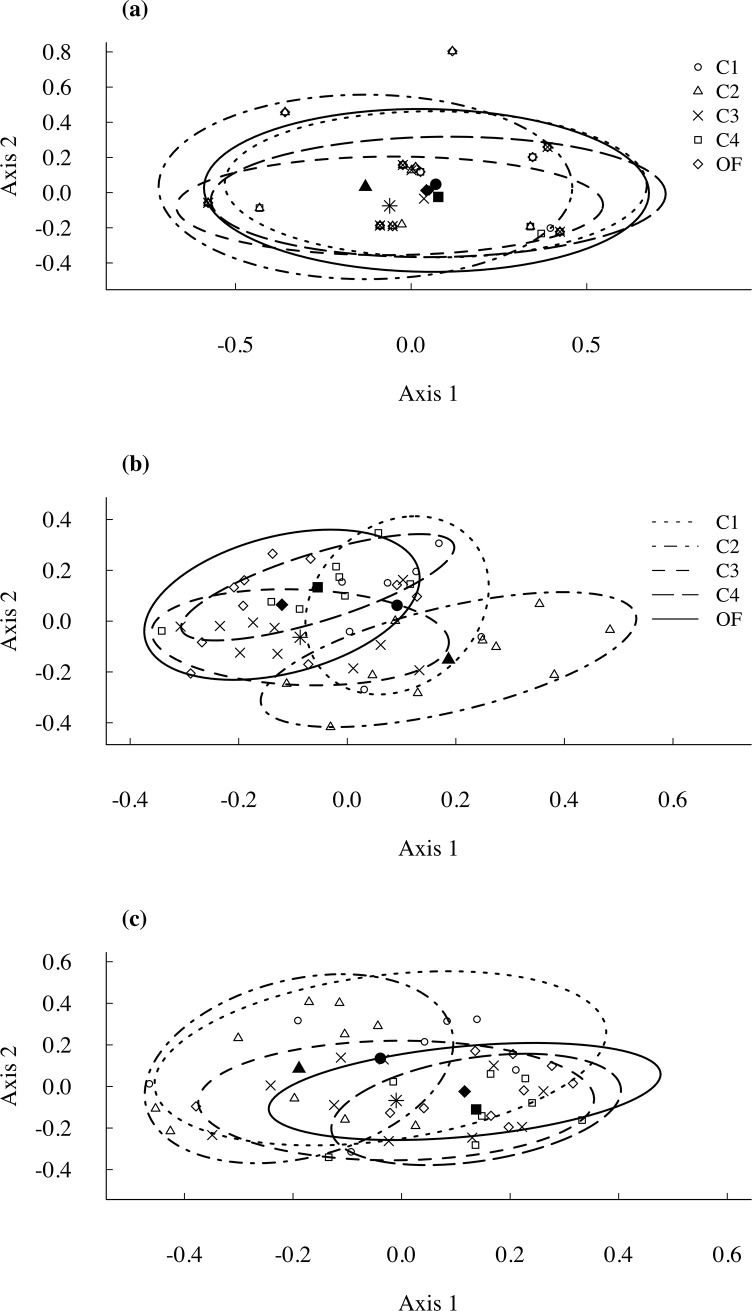
Ordination plots represent dissimilarity in assemblage patterns. Ordination plots of the principal coordinates analyses (based on dissimilarity matrices calculated from Sorensen indices) representing dissimilarity in assemblage patterns among boreal forest age classes for a) small mammals, b) ground-dwelling beetles, and c) flying beetles. The ellipses enclose 80% of the variability in compositional differences accounted for by the first two axes. We used solid lines for old-growth forest, and dashed lines for cut stands of different age after clear-cutting. Age classes: C-1, C-2, C-3, and C-4: 4–20, 21–30, 31–50, and 51–70-years-old clear-cut stands, respectively, OF is for old-growth forest of at least 120-years-old. The solid symbols represent the centroids of the ellipses for each class (C1: circle, C2: triangle, C3: star, C4: square, C5: diamond).

We captured 1449 individuals belonging to 12 species of small mammals across the 53 sites in 2007 and 384 individuals from 12 species across the 34 sites in 2011. PERMANOVA indicated that post-harvest succession had no significant effect on small mammal assemblages (*N* = 75, *F*_4,70_ = 1.19, *P* = 0.316; Fig 2A). The abundance of red-backed voles, however, increased during post-logging forest succession (time-since-logging: regression coefficient ± SE = 0.66 ± 0.08, *P* < 0.001, *N* = 87).

We collected 3894 ground-dwelling beetles belonging to 199 different species (or morphospecies, i.e. “*taxa readily separable by morphological differences that are obvious to individuals without extensive taxonomic training*”, [46]) and 2411 flying beetles to 240 species (or morphospecies). PERMANOVA revealed that species assemblages of both ground-dwelling and flying beetles (based on 58 and 48 species, respectively) varied during post-harvest succession (ground-dwelling beetles: N = 45, *F*_4,40_ = 2.35, *P* = 0.001, Fig 2B; flying beetles: *N* = 45, *F*_4,40_ = 1.76, *P* = 0.003, Fig 2C). Pairwise comparisons following PERMANOVA indicated that ground-dwelling and flying beetle assemblages differed in stands 21–30 years post-harvest from those found in old-growth forests (*P* = 0.004), but no differences were detected between species assemblages in old-growth forests and those of mature stands or recent cuts (*P* > 0.05). PERMDISP indicated that differences in dispersion among the different stand age classes were marginally significant for ground-dwelling beetles (*P* = 0.055; Fig 2B) and significant for flying beetles (*P* = 0.015; Fig 2C). However, this significant difference in the data dispersions for flying beetles was due to differences between two stand age classes (Fig 2C: C2 vs C4; Tukey HSD, *P* = 0.011), but not between old-growth forests and other stands. Consequently, differences revealed by PERMANOVA were attributed mainly to differences in species assemblages across age classes (e.g., centroid differences among stands).

#### Random and non-random patterns of species co-occurrence

**Fig 3:**
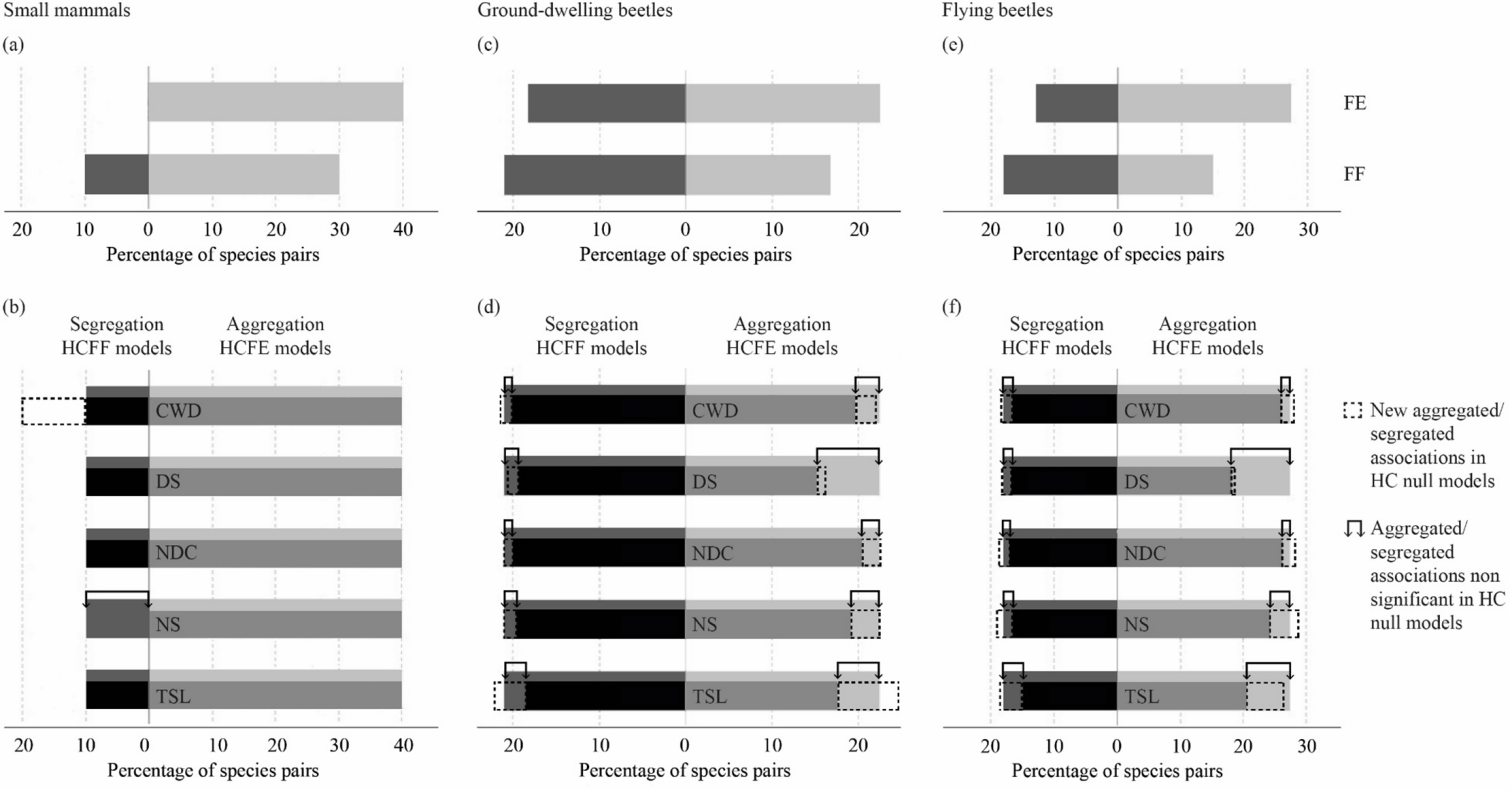
Significantly aggregated or segregated co-occurrence patterns under unconstrained or constrained null models. Percentage of significantly aggregated (grey bars) and segregated (black bars) pairwise species co-occurrences among small mammals (a and b), ground-dwelling beetles (c and d) and flying beetles (e and f) under different unconstrained (a, c, and e, respectively) and habitat-constrained (b, d, and f, respectively) null models (two algorithms: fixed–equiprobable: HCFE and fixed–fixed: HCFF). The significant outcomes under unconstrained FE and FF models are included as background to the constrained models to visualize changes in the significant aggregated and segregated percentage of co-occurrences with the corresponding constrained HCFE and HCFF null models. The significant species co-occurrences from the unconstrained null model that became non-significant under habitat constraint are enclosed between two arrows (indicating pair associations, primarily due to habitat effect). The dotted bars indicate new significant associations under habitat-constrained null models (indicating that the species pairs were segregated within their shared habitat [HCFF] or the newly aggregations were patterns cancelled by the concurrent or opposite effects of habitat heterogeneity indices [HCFE]). HC null models were constrained by either TSL: time-since-logging, or NS: number of different species in the stand, or NDC: number of tree diameter classes in the stand, or DS: dominant tree species in the stand, or CWD: coarse woody debris.

Overall, species co-occurrences could be explained by deterministic processes for at most half of the species pairs (50%, 44% and 46% for small mammals, ground-dwelling and flying beetles, respectively). Among the 10 possible pairs of co-occurrences of small mammal species, a single (10%) negative association (between “lemming and rock vole” and shrews) was detected with the FF unconstrained null models, whereas four (40%) positive associations were detected with the FE unconstrained null models (Fig 3A). We considered a total of 58 ground-dwelling and 48 flying beetle species (i.e., species occurring in at least 5 of the 45 sites). We thus investigated pairwise species co-occurrences for 1653 and 1128 possible pairs of ground-dwelling and flying beetles, respectively. FF unconstrained null models showed that 21% of ground-dwelling beetle pairs and 18% of flying beetle pairs had a significant negative association, whereas FE models showed that 23% ground-dwelling beetle pairs and 27% flying beetle pairs were significantly aggregated (Fig 3C and 3E).

#### Contribution of habitat attributes and interspecific interactions to non-random patterns

Non-random co-occurrence patterns could be partly explained by habitat characteristics (20%, 34% and 41% for small mammals, ground-dwelling and flying beetles, respectively). Using habitat-constrained models, we found that the number of tree and shrub species in the stand explained the only significant segregation that had been previously detected with FF in small mammals. Furthermore, HCFF models that were constrained by coarse woody debris detected a significant segregation between red squirrels and shrews (i.e., negative association within the habitat they shared). Habitat heterogeneity indices (HCFE models) could not explain the four aggregations detected under FE models (i.e., indicating 100% of aggregations are beyond species habitat affinities alone and due to interspecific interactions; Fig 3B).

Results were similar between ground-dwelling and flying species of beetle. For simplicity, we present the results for flying species in parentheses immediately after those for ground-dwelling species. Overall, 83% (71%) of the segregated pairs of beetles that were detected by unconstrained FF models remained significant after accounting for habitat effects using HCFF models (Fig 3D and 3F), indicating that these species pairs are driven mainly by antagonistic interactions. Thus, habitat attributes alone explained only 17% (29%) of the segregations that were detected by unconstrained FF models (area enclosed between arrows; Fig 3D and 3F). Time-since-logging alone could explain 5% (13%) of the segregated patterns (S1 and S2 Figs). HCFF models also detected 102 (92) additional segregated pairs among the possible pairs that were non-significant under the unconstrained (FF) null model (dotted bars in Fig 3D and 3F), indicating that these species were segregated within their shared habitat. Time-since-logging alone explained 42% (32%) of these additional segregations (Fig 3D and 3F). HCFE models also showed that 51% (51%) pairwise aggregations of FE models remained significant when constrained by habitat (Fig 3D and 3F), indicating aggregations beyond expectations of habitat effects alone. Over 49% (49%) of species pairs were explained by habitat alone (area enclosed between arrows; Fig 3D and 3F), but 13% (12%) were explained by the dominant tree species in a stand, whereas time-since-logging mediated 8% (9%) of these (S1 and S2 Figs). Furthermore, HCFE models detected 208 (131) additional significant aggregated pairs (dotted bars in Fig 3D and 3F). These new aggregation patterns were then masked by the differences in affinity for the specific habitat that was considered. Time-since-logging explained 47% (39%) of these new associations, and the number of tree/shrub species in a stand explained 13% (21%).

## Discussion

We showed that tree harvesting influenced the composition of small mammal and beetle communities, with changes becoming detectable 21 to 30 years following disturbance. Overall, our analysis suggests that species co-occur through deterministic processes for about half of the species pairs (50%, 44% and 46% for small mammals, ground-dwelling and flying beetles, respectively), and that deterministic processes were more likely associated with interspecific interactions than with habitat attributes. Our study suggests that the response to clear-cutting varies greatly among taxonomic groups, with some assemblages being more susceptible to disruption (i.e., beetles) from harvesting than others (i.e., small mammals).

Our analysis indicated that small mammal communities remained similar (at least in terms of species occurrence) during forest succession, thereby suggesting that this assemblage was less susceptible to forest disturbance. Consistent with previous studies [29,47], we found that logging reduced the abundance of red-backed voles, which then gradually increased during forest succession. Even if community composition did not vary over time, our results showed that harvesting did affect small mammal communities. Likewise, changes in the relative abundance of other mammal species (deer mice, snowshoe hares *Lepus americanus*) have been recorded during forest succession [29,48]. In contrast to small mammal assemblages, we observed differences in beetle assemblages between 21- to 30-year-old post-harvest stands and old-growth stands. Most old-growth forest beetle species also occurred in recently harvested sites, a pattern that was consistent with observations on carabid and staphylinid beetles in the boreal forest [49,50]. Old-growth species may survive for a certain period of time after harvesting, before drastically decreasing [31]. This response could explain why beetle communities in stands that were 21–30 years post-harvest differed more strongly from those in old-growth forests than assemblages in 5- to 20-year-old post-harvest stands. Similar to our results with small mammals, previous work demonstrated that young stands generally harboured a lower abundance of epigaeic beetles compared to mid-successional stands and mature forests in boreal ecosystems [51].

Our compositional analyses were performed using the most common species because the low number of records of species rarely caught (called rare species in our study) would give us little information on the process structuring the assemblages. The impact of clear-cutting may even be greater than what our analysis revealed, since our approach prevented us from assessing the effect of harvesting on specific rare species, which could be more sensitive to habitat changes than more abundant and conspicuous species [52,53]. Even if conservation generally focuses on species at risk of extinction, our study still has value for conservation since some studies have also underscored the ecological importance of preserving the species most typical of particular ecosystems [38,54]. Moreover, species that are not currently at risk of extinction could become so if current harvesting practices were intensified or changed in a way that affected their dynamics.

Both stochastic and deterministic processes seemed to drive changes in community structure. More specifically, the co-occurrence of about half of the species pairs could be linked to simple random patterns (50%, 56% and 54% for small mammals, ground-dwelling and flying beetles, respectively), which is consistent with the concept that colonization, dispersion and extinction is driven largely by stochastic processes. Accordingly, a study on plant community assembly showed that the relative importance of stochastic and deterministic processes changed during post-fire succession, with stochastic processes driving in early succession and niche-driven dynamics becoming important in later successional stages [55]. Our study demonstrated the importance of stochastic processes such as colonization and dispersal in structuring animal communities. The structure and species composition of vegetation at logged sites (e.g. legacies such as snags and woody debris), together with the landscape setting (e.g. proximity to sources of colonists from old-growth forests) thus can play a significant role in preserving regional biodiversity. The influence of landscape features can be particularly relevant for the preservation of beetle species because structural and compositional habitat heterogeneity effects may extend at least 400 m for flying beetles [2]. Furthermore, mature forests offer a pool of species that could colonize the adjacent harvested stands, thereby influencing beetle communities [56]. Adjacent stands may also enable species to persist in harvested stands by promoting the colonisation through the proximity of source populations or essential habitat elements [57]. Preserving patches of mature forest can increase the chance that small mammal and beetle communities may persist during early successional stages [58–60].

Our results also revealed non-random co-occurrence patterns that could be partly related to habitat characteristics (20%, 34% and 41% for small mammals, ground-dwelling and flying beetles, respectively). Tree and shrub diversity explained the only significant segregations that were detected in small mammals, but none of the four aggregations that were observed among pairs of species. For beetles, the role of habitat characteristics was usually higher for aggregation (49% for both ground-dwelling and flying beetles) than for segregation patterns (17% and 29% for ground-dwelling and flying beetles, respectively). This finding suggested that numerous beetle species have similar habitat affinities, which are mainly related to the state of forest succession and to the tree species dominating in the local stand. Nevertheless, the relationship that species aggregation and segregation share with time-since-logging could reflect the fact that species in young stands have pioneering characteristics favouring generalist species, while species found in old stands could be specialists of old-growth ecosystems [61]. Furthermore, aggregations that are associated with the stand’s dominant tree species can be explained by the fact that multiple beetle species prefer the same tree species, such as black spruce or balsam fir [1,62]. For example, a study that was conducted in this area showed that of 47 saproxylic beetle species collected in snags, 21% of the species were found exclusively in black spruce snags and 36% exclusively in balsam fir [62].

Species–habitat relationships can be strong drivers of animal community structure after disturbance. For example, aggregations and segregations of saproxylic beetle species in post-fire stands are better explained by species–habitat relationships than by interspecific interactions [1]. In post-harvest boreal forest, however, we found that interspecific interactions play an even greater role than habitat attributes in driving non-random co-occurrence patterns. This discrepancy between studies might reflect differences in the effects of harvesting and fire on forest ecosystems, including animal communities. Indeed, it is well known that clear-cutting does not fully mimic post-fire forest dynamics, so distinct species assemblages should be expected [63]. Moreover, wildfires have a more rapid and drastic effect on plants and animals than does logging. Severe fires cause high mortality of macroarthropods with relatively low dispersal abilities [64,65]. Post-fire species communities are thus largely composed of individuals arriving from adjacent stands, either through random dispersal or attracted by specific habitat attributes, such as smoke, heat and infrareds, resulting from a fire event [66,67]. This leads to a community mainly structured by random processes and species-habitat relationships, which are characterized by more specialised species [1,68]. However, the similarity in communities that we observed between recent cuts and old-growth forest stands suggests that species assemblages in young harvested stands would be composed largely of residual populations of mature forest species remaining after harvesting. The presence of residual communities for which resources could be greatly limited after logging, may explain why interspecific interactions play a central role in structuring post-harvest communities. Furthermore, because residual animal populations tend to be much larger in logged than in burned stands [64], random processes structuring communities should play a smaller role in post-logging than in post-fire habitats. Accordingly, about 50% of the co-occurring pairs of species that we observed could be explained by random associations, whereas 70% post-fire saproxylic beetle communities could be linked to random patterns [1].

We found that interspecific interactions could also be strong driver of animal community structure after disturbance, as it explained more than half of non-random species associations (i.e., significant aggregations and segregations). Habitat disturbance, such as logging, can change resource availability (e.g., the intermediate disturbance hypothesis; [69,70] and, in turn, alter the relative roles and outcomes of interspecific interactions in structuring species assemblages (e.g., [17]. In other words, logging may have altered resource availability in such a way that interspecific interactions had a stronger effect in structuring communities than species-habitat relationships in our study. Our results are also congruent with another study showing a trade-off between competitive ability and the capacity to use abundant resources at various stages of forest succession [71]. Moreover, a recent study illustrates that trophic groups of beetles (i.e. predators and decomposers/primary consumers) may have different patterns of recovery during the course of post-logging forest succession [72]. Overall, our analysis reveals variation in response patterns and recovery processes across taxa. The multi-taxa approach allowed us to identify variations in the response of animal communities (i.e., taxa with high or low susceptibility to disturbance), thereby providing a more comprehensive assessment of post-disturbance ecosystem recovery. Notably, we showed that post-logging community assembly was largely driven by species interactions and random dispersal, highlighting the important role of the surrounding landscape to ensure a source of colonizers for cut stands.

## Supporting information

S1 Appendix Null model analysis of species co-occurrence patterns. (DOCX)

S1 Fig The contribution of different habitat attributes for segregated (first number) and aggregated (second number) pairwise species co-occurrences detected by comparing unconstrained null models (FF and FE models, respectively) with habitat-constrained null models (HCFF and HCFE models, respectively) for ground-dwelling beetles. The habitat type explained a total of 17% and 49.5% of segregated (first number) and aggregated (second number) pairwise species co-occurrences, respectively (meaning that interspecific interactions explained 83% and 50.5%, respectively). See text for significant co-occurrences detected by habitat-constrained null models that were regarded as non-significant by unconstrained null models. Values in bold are cited in the results section, the others were considered too marginal to be highlighted in the results section.
(DOCX)

S2 Fig The contribution of different habitat attributes for segregated (first number) and aggregated (second number) in species co-occurrences detected by comparing unconstrained null models (FF and FE models, respectively) with habitat-constrained null models (HCFF and HCFE models, respectively) for flying beetles. The habitat type explained a total of 29.3% and 49.2% of segregated (first number) and aggregated (second number) pairwise species co-occurrences, respectively (meaning that interspecific interactions explained 70.7% and 50.8%, respectively). See text for significant co-occurrences detected by habitat-constrained null models that were regarded as non-significant by unconstrained null models. Values in bold are cited in the results section; the others were considered too marginal to be highlighted in the results section.
(DOCX)
