# Supplementary material for: Temporal dynamics in animal community assembly during post-logging succession in boreal forest
Source: PLoS One. 2018 Sep 20;13(9):e0204445. doi: 10.1371/journal.pone.0204445 (PMC6147515; doi:10.1371/journal.pone.0204445)
Supplement: S1 Appendix — (DOCX) [file pone.0204445.s001.docx]

**Supporting information**

**S1 Appendix. Null model analysis of species co-occurrence patterns**

In our null model analysis, we generated 1000 random matrices for each null model with the *vegan* package in R. To generate FF and HCFF null models, we first applied a quasi-swap algorithm [1] on the species co-occurrence data, which generated random matrices that were independent from the initial matrix and from each other [2]. Because this swapping algorithm produced random matrices that did not have the same probability to be produced (i.e., non-uniform random sampling), each random matrix was then subjected to an ordinary swap algorithm (1000 swaps) to ensure a uniform-random sampling [3] and used the Sørensen Dissimilarity index to evaluate the strength of species pair co-occurrences.

**References**

1. Miklós I, Podani J. Randomization of presence-absence matrices: Comments and new algorithms. Ecology. 2004;85: 86–92. doi:10.1890/03-0101

2. Oksanen J, Blanchet FG, Kindt R, Oksanen MJ, Suggests M. Package “vegan.” Community ecology package Version. 2013;2: 0.

3. Azeria ET, Ibarzabal J, Hébert C. Effects of habitat characteristics and interspecific interactions on co-occurrence patterns of saproxylic beetles breeding in tree boles after forest fire: null model analyses. Oecologia. 2012;168: 1123–1135.
