# Supplementary material for: Temporal dynamics in animal community assembly during post-logging succession in boreal forest
Source: PLoS One. 2018 Sep 20;13(9):e0204445. doi: 10.1371/journal.pone.0204445 (PMC6147515; doi:10.1371/journal.pone.0204445)
Supplement: S1 Fig — The habitat type explained a total of 17% and 49.5% of segregated (first number) and aggregated (second number) pairwise species co-occurrences, respectively (meaning that interspecific interactions explained 83% and 50.5%, respectively). See text for significant co-occurrences detected by habitat-constrained null models that were regarded as non-significant by unconstrained null models. Values in bold are cited in the results section, the others were considered too marginal to be highlighted in the results section. (DOCX) [file pone.0204445.s002.docx]

**Supporting information**


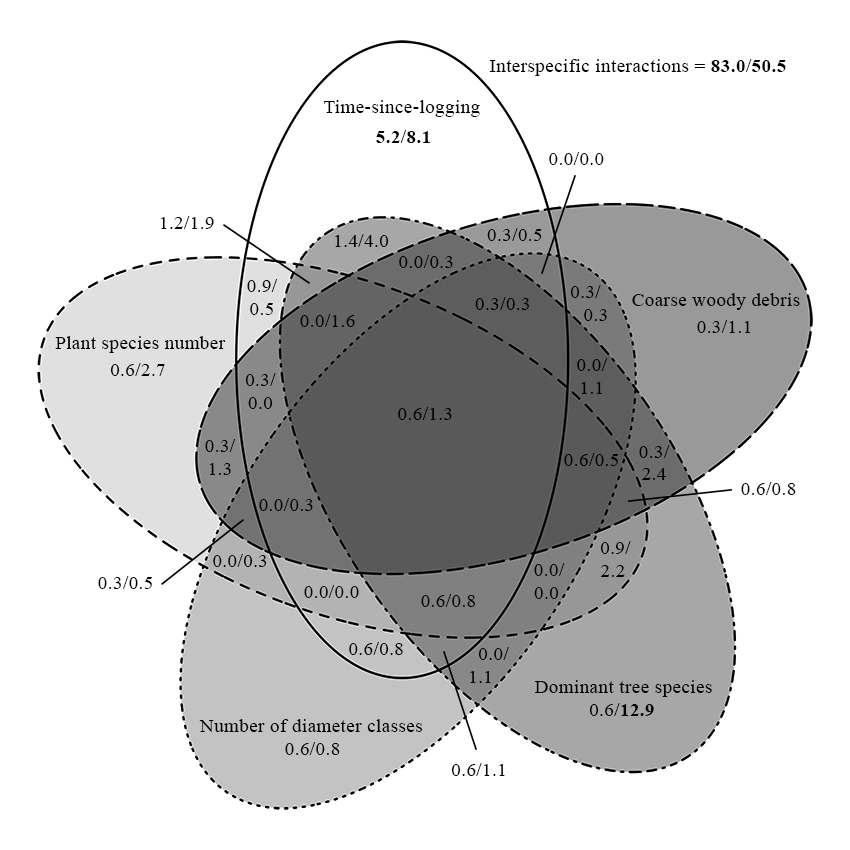


**S1 Fig.** The contribution of different habitat attributes for segregated (first number) and aggregated (second number) pairwise species co-occurrences detected by comparing unconstrained null models (FF and FE models, respectively) with habitat-constrained null models (HCFF and HCFE models, respectively) for ground-dwelling beetles. The habitat type explained a total of 17 % and 49.5 % of segregated (first number) and aggregated (second number) pairwise species co-occurrences, respectively (meaning that interspecific interactions explained 83 % and 50.5 %, respectively). See text for significant co-occurrences detected by habitat-constrained null models that were regarded as non-significant by unconstrained null models. Values in bold are cited in the results section, the others were considered too marginal to be highlighted in the results section.
